# Platelet Indices and Coagulation Parameters in Patients With Complicated and Uncomplicated Type 2 Diabetes Mellitus: A Cross-Sectional Study

**DOI:** 10.7759/cureus.117888

**Published:** 2026-10-13

**Authors:** Mohammad Wasif Manzoor, Shailendra Singh Thakur, Gurvani Rehill, Ashok Panchonia

**Affiliations:** 1 Pathology, Government Medical College Satna, Satna, IND; 2 Pathology, Mahatma Gandhi Memorial Medical College, Indore, IND; 3 Pathology, Tata Trust, Centre for Oncopathology, Mumbai, IND

**Keywords:** coagulation parameters, diabetic complications, mean platelet volume, platelet distribution width, platelet indices, type 2 diabetes mellitus

## Abstract

Background: Vascular complications are a major concern in patients with type 2 diabetes mellitus (T2DM) and may be associated with changes in platelet activity and blood coagulation. This study assessed platelet indices and coagulation parameters in patients with complicated and uncomplicated T2DM.

Methods: This cross-sectional study included 150 participants, equally divided into healthy controls, patients with uncomplicated T2DM, and patients with diabetic complications. Platelet count, mean platelet volume (MPV), platelet distribution width (PDW), platelet large cell ratio (P-LCR), prothrombin time, international normalized ratio, activated partial thromboplastin time, and clotting time were assessed. One-way analysis of variance, Welch's independent-samples t-test, and chi-square test were used for unadjusted comparisons, followed by covariate-adjusted analyses accounting for age across the three groups and for age and HbA1c between the two T2DM groups.

Results: Patients with complicated T2DM had poorer glycemic control, with significantly higher fasting blood sugar, random blood sugar, and glycated hemoglobin levels than those without complications. They also had lower platelet counts and higher MPV, PDW, P-LCR, and coagulation parameters than patients with uncomplicated diabetes and healthy controls. However, exploratory subgroup analysis showed that these differences were not consistent across all individual vascular complications. Among microvascular complications, only PDW was significantly higher in patients with diabetic retinopathy. Among macrovascular complications, selected parameters differed in cerebrovascular disease, while more extensive differences were observed in coronary artery disease.

Conclusion: Platelet indices and coagulation parameters were altered in patients with complicated type T2DM, although the pattern varied across individual complications. These routinely available tests may provide additional information on hemostatic changes in patients with T2DM when interpreted alongside other clinical findings. Further prospective studies are needed to determine their predictive or prognostic value in diabetic complications.

## Introduction

Although diagnosis and treatment of diabetes mellitus (DM) have improved over the years, type 2 DM (T2DM) is still a leading cause of morbidity and mortality because of the onset of long-term microvascular and macrovascular complications [1]. T2DM is one of the most prevalent chronic metabolic diseases in the world and is on the rise as a result of urbanization, poor dietary habits, physical inactivity, obesity, and genetic susceptibility [2].

The hemostatic system is one of several physiological systems that are affected by persistent hyperglycemia. Platelets are central to hemostasis, and alterations in their morphology and function may be a reflection of the development of vascular damage in patients with diabetes [3,4].

Routine complete blood count provides several platelet indices, such as platelet count (PC), mean platelet volume (MPV), platelet distribution width (PDW), and platelet large cell ratio (P-LCR). These parameters are low-cost and easily accessible and have been increasingly investigated as markers of platelet activation. Furthermore, coagulation markers, such as prothrombin time (PT), international normalized ratio (INR), and activated partial thromboplastin time (APTT), offer insights into the coagulation cascade and can be indicative of the hemostatic imbalance in diabetes [5,6]. Larger and more heterogeneous platelets are metabolically more active and have higher thrombotic potential, making MPV, PDW, and P-LCR useful markers of platelet dysfunction in T2DM [7,8].

These tests are simple and inexpensive, are routinely available, and have been shown to be altered in patients with diabetic complications compared to those without complications, suggesting their potential utility as adjunctive markers in the assessment of patients with diabetic complications [9]. However, there is limited evidence comparing these parameters between patients with complicated and uncomplicated T2DM. Therefore, this cross-sectional study aimed to compare platelet indices (PC, MPV, PDW, and P-LCR) and coagulation parameters (PT, INR, APTT, and clotting time) among apparently healthy controls, patients with uncomplicated T2DM, and patients with complicated T2DM. As a secondary exploratory objective, differences according to individual microvascular and macrovascular complications were assessed.

## Materials and methods

This cross-sectional study was conducted in the Department of Medicine in collaboration with the Department of Pathology at Mahatma Gandhi Memorial Medical College and M.Y. Hospital, Indore, Madhya Pradesh, India, after getting approval from the Institutional Ethics Committee (Approval No. EC/MGM/Nov-23/238). The study was conducted from Dec 2023 to May 2025, and informed written consent was obtained from all participants before enrollment.

A total of 150 participants were included in the study, with each group consisting of 50 participants. Eligible participants were recruited using convenience sampling until the target sample size was reached. Group I comprised apparently healthy controls recruited from patients' relatives and the general population. These participants had no known history of DM or other major conditions likely to affect platelet or coagulation parameters and had normal fasting blood glucose, random blood glucose, and HbA1c levels. Group II comprised patients with T2DM without documented complications. The absence of diabetic complications was established from patient history, previous medical records, and assessment by the Department of General Medicine. Group III comprised patients with T2DM with complications identified from established diagnoses documented by the Department of General Medicine and supported by available case-sheet evidence.

Coronary artery disease (CAD) was supported by a physician's diagnosis and available cardiac reports; cerebrovascular disease (CVD) was supported by a documented history of stroke or transient ischemic attack (TIA) and available brain imaging or discharge records; peripheral artery disease (PAD) was supported by vascular assessment and available ankle-brachial index (ABI) or Doppler reports; diabetic nephropathy was supported by urine albumin or kidney function results; diabetic neuropathy was supported by a documented nerve or foot examination; and diabetic retinopathy was supported by an eye examination or ophthalmologist's report. Participants were classified as having no established diabetic complications when no documented diagnosis of any of these complications was present in the medical records. Participants with more than one documented complication were classified under all applicable categories; therefore, the complication categories were not mutually exclusive.

All participants were aged 18 years or older, were willing to participate, and provided written informed consent. Patients with T2DM without established diabetic complications were included in Group II, whereas those with established diabetic complications were included in Group III.

The sample size of 150 participants was calculated by the standard formula for estimating a single population proportion at a 95% confidence level. The minimum sample size was calculated using the reported prevalence of microvascular complications in patients with T2DM in India (41.8%) with an allowable error of 7.8% [10].

Patients with type 1 DM, gestational diabetes, maturity-onset diabetes of the young (MODY), and other specific types of diabetes were excluded. Other exclusion criteria included hematological disorders, bone marrow diseases, malignancies, recent blood transfusion, chemotherapy or antiplatelet therapy, and other conditions likely to independently affect platelet indices or coagulation parameters. To reduce the potential confounding factors, pregnant women, women during menstruation or using oral contraceptive pills, smokers, alcohol users, and patients with anemia (hemoglobin <12 g/dL in females and <14 g/dL in males) were excluded.

A structured data collection proforma was developed by the authors to record demographic and clinical information, medical history, and other relevant study variables; the complete proforma is provided in the Appendix. Venous blood (5 mL) was collected from the antecubital vein under aseptic precautions. Blood intended for platelet indices was collected in 2-mL K3 EDTA non-vacuum blood collection tubes (L-TUBE; Levram Lifesciences Pvt. Ltd., Maharashtra, India), while blood intended for coagulation studies was collected in 1.8-mL non-vacuum blood collection tubes containing 3.2% sodium citrate (S-COLLECT; Sturion Medical Pvt. Ltd., Gujarat, India). Samples were processed within two hours of collection. Platelet samples were analyzed using the BC-3600 three-part automated hematology analyzer (Shenzhen Mindray Bio-Medical Electronics Co., Ltd., Shenzhen, China), while citrated samples were used for coagulation studies. Sample collection and processing details, including collection and analysis times, collection-to-analysis interval, fasting status, sample condition, and any processing deviations, were documented in the study proforma.

PC, MPV, PDW, and P-LCR were measured using a BC-3600 three-part automated hematology analyzer (Shenzhen Mindray Bio-Medical Electronics Co., Ltd., Shenzhen, China). Prothrombin time (PT), international normalized ratio (INR), and activated partial thromboplastin time (APTT) were measured using a Coag4D four-channel semi-automated coagulation analyzer (DIAGON Ltd., Budapest, Hungary). Clotting time was measured by the capillary tube method and reported in minutes. Fasting blood sugar (FBS) and random blood sugar (RBS) were measured using an AccuSure blood glucose monitoring system (MicroGene Diagnostic Systems Pvt. Ltd., New Delhi, India). Glycated haemoglobin (HbA1c) was measured using the D-10 Hemoglobin Testing System (Bio-Rad Laboratories, Hercules, CA) by ion-exchange high-performance liquid chromatography (HPLC). Automated hematological results were confirmed by peripheral blood smear examination in all cases.

Continuous variables were summarized using mean and standard deviation, while categorical variables were summarized using frequencies and percentages. Data were entered into Microsoft Excel (Microsoft® Corp., Redmond, WA) and analyzed using R version 4.6.0 (R Foundation for Statistical Computing, Vienna, Austria). There were no missing data for the variables included in the analysis; therefore, no imputation was performed. The distribution of continuous variables was assessed for normality before inferential analysis. For comparisons of continuous variables across the three independent study groups (healthy controls, T2DM without complications, and T2DM with complications), one-way analysis of variance (ANOVA) was used. For comparisons between two independent groups, such as patients with and without a specific diabetic complication, Welch's independent-samples t-test was used when the continuous variables satisfied the assumptions for parametric testing. Categorical variables, including sex and age-group distribution, were compared using the Pearson chi-square test. A p-value < 0.05 was considered statistically significant.

The primary comparison among the three study groups is now explicitly stated in the Statistical Analysis section, while individual complication-specific comparisons are identified as secondary exploratory analyses. For the age-adjusted analysis, a covariate-adjusted general linear model/analysis of covariance (ANCOVA) framework was used, with study group (healthy controls, T2DM without complications, and T2DM with complications) as the categorical grouping variable and age as the covariate. Adjusted estimated marginal means were obtained for each group, and pairwise comparisons were performed using Tukey adjustment, with adjusted differences and 95% confidence intervals reported. For the age- and HbA1c-adjusted analysis, the comparison was restricted to the two T2DM groups, with complication status (T2DM without complications vs. T2DM with complications) as the grouping variable and age and HbA1c as covariates. Adjusted means, adjusted between-group differences, 95% confidence intervals, and p-values were reported.

Analyses according to individual diabetic complications were considered exploratory subgroup analyses and were not adjusted for multiple comparisons because of the multiple comparisons and small subgroup sizes. Accordingly, the corresponding p-values were interpreted as nominal and hypothesis-generating.

## Results

The mean age was 50.4 ± 16.1 years in the DM without complications group, 47.2 ± 8.4 years in the DM with complications group, and 47.3 ± 17.6 years among controls, with no statistically significant difference (p = 0.468). However, age-group distribution differed significantly across the groups (p < 0.001). The distribution of male and female participants was comparable, with no significant difference in sex distribution (p = 0.785) (Table 1).

**Table 1:** Baseline demographic and clinical characteristics of the study participants Data are presented as mean ± SD for age and as n (%) for age-group and sex distributions. One-way ANOVA was used to compare mean age among the three study groups, and Pearson's chi-square test was used to compare age-group and sex distributions. p < 0.05 was considered statistically significant. ANOVA: analysis of variance; DM: diabetes mellitus; SD: standard deviation

Variable	DM without complications (n=50)	DM with complications (n=50)	Control (n=50)	Test statistic	p-value
Age (years), Mean ± SD	50.4 ± 16.1	47.2 ± 8.4	47.3 ± 17.6	F = 0.764	0.468
Age group, n (%)					
18-40 years	21 (42.0)	15 (30.0)	11 (22.0)	χ² = 25.825	<0.001
41-60 years	14 (28.0)	20 (40.0)	37 (74.0)		
61-80 years	15 (30.0)	15 (30.0)	2 (4.0)		
Gender, n (%)					
Male	26 (52.0)	29 (58.0)	26 (52.0)	χ² = 0.483	0.785
Female	24 (48.0)	21 (42.0)	24 (48.0)		

RBS, FBS, and HbA1c levels were also higher in the complication group, with statistically significant differences observed for all three parameters (all p < 0.001) (Table 2).

**Table 2:** Distribution of clinical characteristics of patients with type 2 diabetes mellitus according to complication status of the study participants Data are presented as mean ± SD. Welch's independent-samples t-test was used to compare patients with and without complications. P < 0.05 was considered statistically significant. DM: diabetes mellitus, FBS: fasting blood sugar, HbA1c: glycated hemoglobin, RBS: random blood sugar, SD: standard deviation

Variable	DM without complications (n=50)	DM with complications (n=50)	t-value	p-value
RBS	351.6 ± 67.7	521.2 ± 29.3	16.254	<0.001
FBS	237.9 ± 53.4	367.5 ± 36.4	14.180	<0.001
HbA1c	9.78 ± 3.35	12.77 ± 0.84	6.103	<0.001

Macrovascular and microvascular complications were observed in patients with complicated T2DM. Coronary artery disease was the most common macrovascular complication, whereas diabetic nephropathy was the most frequent microvascular complication (Table 3).

**Table 3:** Distribution of microvascular and macrovascular complications among patients with type 2 diabetes mellitus with complications (n=50) Data are presented as frequency (n) and percentage (%). Participants could have more than one documented complication; therefore, complication categories were not mutually exclusive, and frequencies may exceed the total number of participants. CAD: coronary artery disease; CVD: cerebrovascular disease; PAD: peripheral artery disease

Type	Complication	Frequency	Percent (%)
Microvascular Complications	Diabetic nephropathy	12	24.0
	Diabetic neuropathy	7	14.0
	Diabetic retinopathy	10	20.0
Macrovascular Complications	CVD	8	16.0
	PAD	10	20.0
	CAD	14	28.0

There were statistically significant differences in all platelet indices and coagulation parameters among the three study groups. Patients with complicated T2DM had lower platelet counts and higher platelet indices (MPV, PDW, and P-LCR), as well as higher PT, INR, APTT, and clotting time, compared with patients without complications and healthy controls (Table 4).

**Table 4:** Comparison of platelet indices and coagulation parameters among the study groups Data are presented as mean ± SD. One-way ANOVA was used to compare platelet indices and coagulation parameters among the three study groups. P < 0.05 was considered statistically significant. ANOVA: analysis of variance, APTT: activated partial thromboplastin time, DM: diabetes mellitus, fL: femtoliter, INR: international normalized ratio, MPV: mean platelet volume, P-LCR: platelet large cell ratio, PDW: platelet distribution width, PT: prothrombin time, SD: standard deviation, T2DM: type 2 diabetes mellitus

Parameter	DM without complications (n=50)	DM with complications (n=50)	Control (n=50)	F-value	p-value
Platelet count (10⁹/L)	222.0 ± 12.2	194.0 ± 12.0	248.0 ± 7.87	307.461	<0.001
MPV (fL)	9.27 ± 0.30	10.30 ± 0.37	8.85 ± 0.21	295.255	<0.001
PDW (%)	13.4 ± 0.41	14.7 ± 0.60	13.0 ± 0.24	189.471	<0.001
P-LCR (%)	25.0 ± 1.00	28.7 ± 1.16	22.4 ± 0.61	552.491	<0.001
PT (sec)	13.5 ± 0.28	14.5 ± 0.57	13.1 ± 0.24	176.006	<0.001
INR	1.01 ± 0.02	1.14 ± 0.04	0.986 ± 0.02	355.739	<0.001
APTT (sec)	31.4 ± 0.97	36.4 ± 1.44	30.0 ± 0.74	473.499	<0.001
Clotting time (mins)	6.57 ± 0.19	7.68 ± 0.55	6.24 ± 0.14	237.421	<0.001

Exploratory subgroup analyses showed that platelet indices and coagulation parameters did not differ significantly according to diabetic nephropathy or diabetic neuropathy status. Among patients with diabetic retinopathy, PDW was higher in those with retinopathy than in those without retinopathy (15.10 ± 0.51 vs. 14.55 ± 0.58; nominal p = 0.009). Given the small sample sizes of several complication subgroups, these findings should be interpreted cautiously (Table 5).

**Table 5:** Exploratory comparison of platelet indices and coagulation parameters according to microvascular complications Given the small sample sizes of several complication subgroups, these analyses were considered exploratory. P-values are nominal and were not adjusted for multiple comparisons. Findings should therefore be interpreted cautiously. For each individual complication, "No" indicates absence of that specific complication and does not imply absence of other complications. Welch's independent-samples t-test was used to compare platelet indices and coagulation parameters between patients with and without each microvascular complication. P < 0.05 was considered statistically significant. APTT: activated partial thromboplastin time, INR: international normalized ratio, MPV: mean platelet volume, P-LCR: platelet large cell ratio, PDW: platelet distribution width, PT: prothrombin time, SD: standard deviation

Parameter	Diabetic nephropathy (Mean ± SD)				Diabetic neuropathy (Mean ± SD)				Diabetic retinopathy (Mean ± SD)			
	No (n=38)	Yes (n=12)	t-value	p-value	No (n=43)	Yes (n=7)	t-value	p-value	No (n=40)	Yes (n=10)	t-value	p-value
Platelet count	193.31 ± 12.45	196.68 ± 10.45	-0.93	0.362	193.14 ± 12.14	200.13 ± 9.64	-1.711	0.12	194.01 ± 12.25	194.55 ± 11.48	-0.132	0.897
MPV	10.26 ± 0.34	10.30 ± 0.45	-0.298	0.77	10.26 ± 0.37	10.31 ± 0.36	-0.363	0.726	10.23 ± 0.38	10.43 ± 0.27	-1.954	0.065
PDW	14.65 ± 0.59	14.68 ± 0.68	-0.115	0.91	14.64 ± 0.63	14.74 ± 0.49	-0.485	0.638	14.55 ± 0.58	15.10 ± 0.51	-3.002	0.009
P-LCR	28.52 ± 1.16	29.18 ± 1.08	-1.819	0.084	28.65 ± 1.20	28.89 ± 0.99	-0.575	0.579	28.64 ± 1.17	28.83 ± 1.17	-0.453	0.658
PT	14.50 ± 0.55	14.55 ± 0.65	-0.242	0.812	14.51 ± 0.59	14.56 ± 0.45	-0.273	0.791	14.46 ± 0.57	14.74 ± 0.49	-1.586	0.133
INR	1.138 ± 0.042	1.139 ± 0.052	-0.061	0.952	1.139 ± 0.046	1.133 ± 0.032	0.461	0.654	1.134 ± 0.043	1.154 ± 0.046	-1.223	0.242
APTT (sec)	36.36 ± 1.40	36.55 ± 1.60	-0.367	0.718	36.39 ± 1.49	36.49 ± 1.17	-0.186	0.856	36.26 ± 1.43	36.99 ± 1.39	-1.48	0.161
Clotting time (mins)	7.69 ± 0.51	7.66 ± 0.70	0.143	0.888	7.67 ± 0.57	7.74 ± 0.46	-0.365	0.723	7.62 ± 0.55	7.93 ± 0.52	-1.675	0.115

Exploratory subgroup analyses showed variation in platelet indices and coagulation parameters according to individual macrovascular complications. Among patients with CVD, nominally significant differences were observed for MPV, PT, and clotting time, whereas no nominally significant differences were observed according to PAD status. Among patients with CAD, nominally significant differences were observed for MPV, PDW, P-LCR, PT, INR, APTT, and clotting time, while platelet count did not differ significantly. Given the small sample sizes of several complication subgroups and the number of comparisons performed, these findings should be interpreted cautiously (Table 6).

**Table 6:** Exploratory comparison of platelet indices and coagulation parameters according to macrovascular complications Data are presented as mean ± SD. Welch's independent-samples t-test was used to compare platelet indices and coagulation parameters between patients with and without each macrovascular complication. P < 0.05 was considered statistically significant. Given the small sample sizes of several complication subgroups, these analyses were considered exploratory. P-values are nominal and were not adjusted for multiple comparisons. Findings should therefore be interpreted cautiously. For each complication, "No" indicates absence of that specific complication and does not imply absence of other complications. APTT: activated partial thromboplastin time, CAD: coronary artery disease, CVD: cerebrovascular disease, fL: femtoliter, INR: international normalized ratio, MPV: mean platelet volume, P-LCR: platelet large cell ratio, PAD: peripheral artery disease, PDW: platelet distribution width, PT: prothrombin time, SD: standard deviation

Parameter	CVD (Mean ± SD)				PAD (Mean ± SD)				CAD (Mean ± SD)			
	No (n=42)	Yes (n=8)	t-value	p-value	No (n=40)	Yes (n=10)	t-value	p-value	No (n=36)	Yes (n=14)	t-value	p-value
Platelet count (10⁹/L)	195 ± 12.3	192 ± 10.7	0.59	0.568	194 ± 12.4	193 ± 10.8	0.361	0.723	194 ± 12.0	195 ± 12.3	-0.195	0.847
MPV (fL)	10.3 ± 0.37	9.99 ± 0.24	3.32	0.0049	10.3 ± 0.36	10.3 ± 0.41	-0.018	0.986	10.2 ± 0.37	10.5 ± 0.24	-3.474	0.0013
PDW (%)	14.7 ± 0.61	14.4 ± 0.58	1.153	0.275	14.7 ± 0.58	14.6 ± 0.71	0.441	0.667	14.5 ± 0.59	15.1 ± 0.42	-3.95	<0.001
P-LCR (%)	28.7 ± 1.25	28.5 ± 0.58	0.766	0.452	28.7 ± 1.17	28.7 ± 1.21	-0.089	0.931	28.4 ± 1.06	29.4 ± 1.10	-3.031	0.0059
PT (sec)	14.6 ± 0.58	14.2 ± 0.37	2.24	0.0413	14.5 ± 0.56	14.4 ± 0.62	0.419	0.682	14.4 ± 0.53	14.9 ± 0.48	-3.47	0.0018
INR	1.14 ± 0.044	1.12 ± 0.040	1.694	0.12	1.14 ± 0.045	1.13 ± 0.044	0.677	0.509	1.13 ± 0.044	1.16 ± 0.038	-2.389	0.0242
APTT (sec)	36.5 ± 1.47	35.9 ± 1.21	1.307	0.217	36.5 ± 1.43	36.2 ± 1.52	0.483	0.637	35.9 ± 1.27	37.7 ± 1.01	-5.154	<0.001
Clotting time (mins)	7.75 ± 0.55	7.31 ± 0.42	2.56	0.0249	7.70 ± 0.54	7.60 ± 0.61	0.483	0.637	7.49 ± 0.51	8.18 ± 0.30	-5.942	<0.001

After adjustment for age, platelet count remained progressively lower across controls, T2DM without complications, and T2DM with complications, with adjusted means of 248.06, 221.83, and 194.15, respectively. Conversely, MPV increased from 8.85 to 9.26 to 10.27, while P-LCR increased from 22.39 to 24.97 to 28.68 across the three groups. Among coagulation parameters, PT increased from 13.09 to 13.51 to 14.52, and APTT from 30.04 to 31.38 to 36.42, respectively. All pairwise adjusted comparisons were statistically significant (all p < 0.001; Table 7).

**Table 7:** Age-adjusted comparison of platelet indices and coagulation parameters across the three study groups Values are adjusted for age. Pairwise comparisons were performed using estimated marginal means with Tukey adjustment. The adjusted means and CIs are directly from the final R analysis. MPV: mean platelet volume, PDW: platelet distribution width, P-LCR: platelet-large cell ratio, PT: prothrombin time, INR: international normalized ratio, APTT: activated partial thromboplastin time, CI: confidence interval

Parameter	Control, adjusted mean (95% CI)	DM without complications, adjusted mean (95% CI)	DM with complications, adjusted mean (95% CI)	Pairwise adjusted difference	95% CI	p-value
Platelet count	248.06 (245.02-251.11)	221.83 (218.77-224.89)	194.15 (191.10-197.20)	Control vs DM without: 26.23	21.05-31.41	<0.001
				Control vs DM with: 53.91	48.75-59.07	<0.001
				DM without vs DM with: 27.68	22.50-32.86	<0.001
MPV	8.85 (8.77-8.93)	9.26 (9.18-9.35)	10.27 (10.18-10.35)	Control vs DM without: −0.41	−0.56 to −0.27	<0.001
				Control vs DM with: −1.42	−1.56 to −1.28	<0.001
				DM without vs DM with: −1.00	−1.15 to −0.86	<0.001
PDW	13.00 (12.88-13.12)	13.40 (13.28-13.53)	14.66 (14.53-14.78)	Control vs DM without: −0.40	−0.61 to −0.19	<0.001
				Control vs DM with: −1.66	−1.87 to −1.45	<0.001
				DM without vs DM with: −1.25	−1.47 to −1.04	<0.001
P-LCR	22.39 (22.12-22.66)	24.97 (24.70-25.24)	28.68 (28.42-28.95)	Control vs DM without: −2.58	−3.03 to −2.13	<0.001
				Control vs DM with: −6.29	−6.74 to −5.84	<0.001
				DM without vs DM with: −3.71	−4.17 to −3.26	<0.001
PT	13.09 (12.98-13.20)	13.51 (13.40-13.62)	14.52 (14.41-14.62)	Control vs DM without: −0.42	−0.61 to −0.24	<0.001
				Control vs DM with: −1.43	−1.61 to −1.24	<0.001
				DM without vs DM with: −1.00	−1.19 to −0.82	<0.001
INR	0.99 (0.98-0.99)	1.01 (1.01-1.02)	1.14 (1.13-1.15)	Control vs DM without: −0.028	−0.042 to −0.013	<0.001
				Control vs DM with: −0.152	−0.167 to −0.138	<0.001
				DM without vs DM with: −0.125	−0.139 to −0.110	<0.001
APTT (sec)	30.04 (29.74-30.35)	31.38 (31.08-31.68)	36.42 (36.12-36.72)	Control vs DM without: −1.33	−1.85 to −0.82	<0.001
				Control vs DM with: −6.37	−6.89 to −5.86	<0.001
				DM without vs DM with: −5.04	−5.55 to −4.53	<0.001
Clotting time (mins)	6.24 (6.15-6.34)	6.57 (6.47-6.66)	7.68 (7.59-7.78)	Control vs DM without: −0.33	−0.49 to −0.16	<0.001
				Control vs DM with: −1.44	−1.61 to −1.28	<0.001
				DM without vs DM with: −1.12	−1.28 to −0.95	<0.001

After adjustment for age and HbA1c, patients with T2DM with complications had a lower adjusted platelet count than those without complications (193.31 vs. 222.71), while MPV (10.25 vs. 9.28), PDW (14.62 vs. 13.45), and P-LCR (28.69 vs. 24.97) were higher. Coagulation parameters were also higher in patients with complications, including PT (14.48 vs. 13.55), INR (1.14 vs. 1.02), APTT (36.32 vs. 31.49), and clotting time (7.64 vs. 6.61). All adjusted comparisons were statistically significant (all p < 0.001; Table 8).

**Table 8:** Age- and HbA1c-adjusted comparison of platelet indices and coagulation parameters between diabetic patients with and without complications Interpretation values are adjusted for age and HbA1c. Adjusted means, between-group differences, 95% confidence intervals, and p-values were obtained from the final R analysis. The adjusted difference represents the difference between the adjusted mean for T2DM without complications and T2DM with complications. Adjusted for age and HbA1c. Compared with patients with T2DM without complications, patients with complicated T2DM had lower adjusted platelet count and higher adjusted MPV, PDW, P-LCR, PT, INR, APTT, and clotting time. All adjusted 95% CIs excluded the null value, and all comparisons were statistically significant (p < 0.001). MPV: mean platelet volume, PDW: platelet distribution width, P-LCR: platelet-large cell ratio, PT: prothrombin time, INR: international normalized ratio, APTT: activated partial thromboplastin time, CI: confidence interval, HbA1c: glycated hemoglobin

Parameter	DM without complications, adjusted mean (95% CI)	DM with complications, adjusted mean (95% CI)	Adjusted difference*	95% CI	p-value
Platelet count	222.71 (218.99-226.42)	193.31 (189.60-197.02)	29.4	23.71-35.08	<0.001
MPV	9.28 (9.18-9.39)	10.25 (10.15-10.35)	−0.97	−1.13 to −0.81	<0.001
PDW	13.45 (13.29-13.61)	14.62 (14.46-14.77)	−1.17	−1.41 to −0.92	<0.001
P-LCR	24.97 (24.63-25.31)	28.69 (28.35-29.03)	−3.72	−4.24 to −3.20	<0.001
PT	13.55 (13.42-13.69)	14.48 (14.34-14.62)	−0.93	−1.14 to −0.72	<0.001
INR	1.02 (1.01-1.03)	1.14 (1.13-1.15)	−0.12	−0.14 to −0.10	<0.001
APTT (sec)	31.49 (31.11-31.86)	36.32 (35.94-36.70)	−4.84	−5.41 to −4.26	<0.001
Clotting time (mins)	6.61 (6.49-6.74)	7.64 (7.51-7.77)	−1.03	−1.22 to −0.83	<0.001

## Discussion

The present study demonstrated significant alterations in platelet indices and coagulation parameters among patients with T2DM, particularly those with vascular complications. Patients with complicated T2DM had poorer glycemic control than those without complications, consistent with the observed association between glycemic status and diabetic vascular complications.

The significantly higher RBS, FBS, and HbA1c levels observed in the complicated T2DM group support the association between poor glycemic control and diabetic vascular complications. Chronic hyperglycemia may contribute to endothelial injury, oxidative stress, and progressive vascular damage. Previous studies by Khanna et al., Dwivedi et al., and Jhavar et al. have also reported an association between longer duration of diabetes and an increased occurrence or severity of diabetic complications [6,11,12]. Similarly, the relationship between poor glycemic control and diabetic vascular disease observed in the present study is consistent with findings from the UK Prospective Diabetes Study, which demonstrated that improved glycemic control reduces the risk of microvascular complications [13].

In the present study, coronary artery disease was the most common macrovascular complication, while diabetic nephropathy was the most common microvascular complication. Differences in the distribution of complications across studies may be influenced by variations in study populations, glycemic status, and screening practices.

Patients with complicated T2DM had lower platelet count, while MPV, PDW, and P-LCR were significantly elevated. This pattern may be consistent with altered platelet size distribution and turnover in patients with diabetic complications; however, platelet indices are indirect measures and do not directly assess platelet activation or function. The higher MPV and PDW in diabetic patients, especially those with complications, may be related to the greater metabolic and thrombotic potential of larger platelets [6,14]. However, the cross-sectional design does not permit determination of whether these platelet alterations contribute to, or result from, vascular complications. Samaddar et al. and Bhatta et al., however, did not find any significant difference in platelet count, indicating that platelet count might not be as reliable as functional platelet indices [15,16].

Patients with complicated T2DM had higher PT, INR, APTT, and clotting time than patients without complications and healthy controls, indicating alterations in hemostatic parameters. These changes may be related to endothelial dysfunction, chronic inflammation, and glycation-related alterations in coagulation proteins, which can contribute to an altered hemostatic state [3].

Importantly, the observed differences in platelet indices and coagulation parameters persisted after adjustment for age, supporting the robustness of the group-wise differences despite differences in age distribution. Furthermore, after adjustment for age and HbA1c, patients with T2DM with complications continued to show lower platelet counts and higher MPV, PDW, P-LCR, PT, INR, APTT, and clotting time compared with those without complications, suggesting that these differences were not fully explained by age or HbA1c.

Exploratory subgroup analyses showed that these alterations were not uniform across individual diabetic complications. Among microvascular complications, only PDW differed nominally according to retinopathy status, whereas no nominally significant differences were observed according to nephropathy or neuropathy status. Among macrovascular complications, nominally significant differences were observed for selected parameters in patients with CVD and more extensively in those with CAD, while no nominally significant differences were observed according to PAD status. Given the small subgroup sizes and multiple comparisons, these findings should be considered exploratory and interpreted cautiously.

Limitations

This study has several limitations. Its cross-sectional design precludes establishing a temporal or causal relationship between alterations in platelet indices and coagulation parameters and the development of diabetic complications. The study was conducted at a single tertiary care center with a relatively small sample size, and recruitment of participants from hospital-based services, with controls drawn from patients' relatives and the general population, may have introduced selection bias and limited generalizability. Although mean age was comparable among the three groups, the distribution across age categories differed significantly (p < 0.001), with a higher proportion of controls aged 41-60 years and a higher proportion of participants with and without diabetic complications aged 61-80 years, which may represent an additional source of confounding. Age-adjusted and age- and HbA1c-adjusted comparisons were performed. Treatment information was recorded in the study proforma but was not incorporated into the adjusted analyses. Inpatient/outpatient status was not captured as a dedicated study variable and therefore could not be included in the adjusted analyses. These factors have been acknowledged as potential sources of residual confounding.

The individual microvascular and macrovascular complication analyses were exploratory, involved small subgroups, and included overlapping complication categories because some participants had more than one documented complication. No formal adjustment for multiple comparisons was applied to these exploratory complication-specific analyses, which may have increased the risk of type I error; therefore, these findings should be interpreted cautiously and considered hypothesis-generating. Although standardized laboratory procedures and timely sample processing were used, unmeasured pre-analytical and assay-related variability cannot be completely excluded. Additionally, platelet indices are indirect hematological measures and should not be interpreted as direct measures of platelet activation or function. The absence of longitudinal follow-up also prevented assessment of whether these hematological and coagulation changes precede or predict the development of diabetic complications. Larger, multicenter prospective studies with longitudinal follow-up are needed to confirm these findings and evaluate their clinical and predictive relevance.

## Conclusions

This study found notable differences in platelet indices and coagulation parameters among patients with T2DM, particularly in those with diabetic complications. Compared with patients without complications and healthy controls, patients with complicated T2DM had lower platelet counts and higher MPV, PDW, P-LCR, PT, INR, APTT, and clotting time. However, these differences were not seen consistently across all individual microvascular and macrovascular complications. As these tests are routinely available and relatively inexpensive, they may provide additional information about hemostatic changes in patients with T2DM when interpreted alongside other clinical findings. Further prospective studies are needed to determine whether these parameters have predictive or prognostic value in diabetic complications.

## Appendices

**Table 9:** Data collection proforma for the assessment of platelet indices and coagulation parameters in patients with type 2 diabetes mellitus Values are recorded in the units specified. Yes/No responses indicate the presence or absence of the specified condition or criterion; Not applicable indicates that the item does not apply to the participant. Diagnostic evidence/remarks should be documented where applicable. BMI: body mass index, DM: diabetes mellitus, EDTA: ethylenediaminetetraacetic acid, HbA1c: glycated hemoglobin, IPD: inpatient department, OPD: outpatient department

Section	Variable	Response/Result
Participant Information	Participant Study ID	
	Hospital/OPD/IPD No.	
	Date of Enrolment	
	Date of Sample Collection	
A. Study Group	Study group	DM without complication / DM with complication / Healthy control
B. Eligibility	Written informed consent obtained	Yes / No
	Age ≥18 years	Yes / No
	Type 2 diabetes mellitus	Yes / No / Not applicable
C. Sociodemographic Details	Name/Initials	
	Age	______ years
	Age group	18-40 / 41-60 / 61-80 years
	Gender	Male / Female / Other
	Height	______ cm
	Weight	______ kg
	BMI	______ kg/m²
	Blood pressure	______ / ______ mmHg
D. Diabetes History	Diabetes present	Yes / No
	Date/Year of diagnosis	
	Duration of diabetes	______ years ______ months
	Current treatment	Diet and lifestyle modification / Oral antidiabetic drugs / Insulin / Oral drugs and insulin
E. Macrovascular Complications	Cerebrovascular disease	No / Yes Diagnostic evidence/remarks: __________________
	Peripheral artery disease	No / Yes Diagnostic evidence/remarks: __________________
	Coronary artery disease	No / Yes Diagnostic evidence/remarks: __________________
	Other	No / Yes Diagnostic evidence/remarks: __________________
F. Microvascular Complications	Diabetic nephropathy	No / Yes Diagnostic evidence/remarks: __________________
	Diabetic neuropathy	No / Yes Diagnostic evidence/remarks: __________________
	Diabetic retinopathy	No / Yes Diagnostic evidence/remarks: __________________
	Other	No / Yes Diagnostic evidence/remarks: __________________
	Final complication status	No documented complication / One or more complications present
G. Sample Collection and Processing	Volume of venous blood collected	______ mL
	Collection time	
	Analysis time	
	Collection-to-analysis interval	______ minutes
	Fasting status	Fasting / Non-fasting
	EDTA sample received	Yes / No
	Citrate sample received	Yes / No
	Sample condition	Acceptable / Clotted / Haemolysed / Underfilled / Other: __________
	Sample analysed after two hours	Yes / No
	Deviation, if any	
H. Glycaemic and Haematological Investigations	Fasting blood sugar	______ mg/dL
	Random blood sugar	______ mg/dL
	Postprandial blood sugar	______ mg/dL
	HbA1c	______ %
	Haemoglobin	______ g/dL
	Peripheral smear findings	
I. Platelet Parameters	Platelet count	______ ×10^9^/L
	Mean platelet volume	______ fL
	Platelet distribution width	______ %/analyser unit
	Platelet large-cell ratio	______ %
	Instrument used	
J. Coagulation Parameters	Prothrombin time	______ seconds
	International normalized ratio	______ ratio
	Activated partial thromboplastin time	______ seconds
	Clotting time	
	Instrument/Method used	
Data Collection	Data collected by	
	Signature	
	Date	
